# Evaluation of Satisfaction and Axial Rigidity with Titan XL Cylinders

**DOI:** 10.1155/2012/896070

**Published:** 2012-09-11

**Authors:** Gerard D. Henry, Caroline Jennermann, J. Francois Eid

**Affiliations:** ^1^Regional Urology, Department of Urology, 255 Bert Kouns, Shreveport, LA 71199, USA; ^2^Advanced Urological Care, P.C., Department of Urology, 435 East 63rd Street, New York, NY 10065, USA

## Abstract

The inflatable penile prosthesis (IPP) has high patient satisfaction rates and good mechanical reliability rates in multiple studies. The number one patient compliant at six months is penile length. Recently, new technique for aggressive sizing of the cylinders has been published on in the literature. One IPP company has produced a new product that has longer length cylinders (XL) than those available. However, traditionally long cylinders were felt to lack axial rigidity. Therefore, a prospective, multicenter, central IRB-approved, monitored study was performed on the new product to address these concerns. At 2 centers, a total of 17 patients underwent surgical implantation of these new XL cylinders. These patients were questioned for patient satisfaction and tested for axial rigidity using a Fastsize Erectile Quality Monitor. The results showed excellent patient satisfaction rates and great axial rigidity with the Fastsize Erectile Quality Monitor. The XL cylinders appear to give the IPP surgeon the ability to use longer cylinders with good patient satisfaction and great axial rigidity.

## 1. Introduction

Scott et al., [1] who introduced the first inflatable penile prosthesis (IPP), first suggested implantation of serially larger cylinders to enlarge the penis. Although overall patient satisfaction with the IPP is high [2, 3] the most common complaint among IPP patients 6 months postoperatively is penile shortening [4, 5]. Recently, Henry et al. reported a new length measurement technique that resulted in more patients being implanted with larger cylinders, with satisfaction rates comparable to standard measurement techniques [6]. Longer cylinders allow surgeons the option of using more of the cylinder rather than more of the rear-tip extenders (RTEs), giving the patient a more natural appearing penis in erection and in flaccidity. The traditional Titan IPP line offers cylinder sizes up to 22 cm, but recently larger sizes (24 cm, 26 cm, and 28 cm) have been reintroduced. Titan XL large cylinders, made of bioflex, are developed to meet the needs of those subjects with larger penis sizes and revision subjects needing a cylinder size greater than 22 cm. However, in the past, concerns of the lack of axial rigidity have been bought up when using longer cylinders and whether this would affect patient satisfaction.

Therefore, a prospective, multicenter, central IRB-approved, monitored study was performed on this new product to address these concerns. The primary objective of the study was to assess the rigidity of the Titan IPP cylinders ≥24 cm using the Fastsize Erectile Quality Monitor (EQM) postimplant. Secondary objectives include assessment of subjects' and investigators' perception of sufficient rigidity for sexual function; subject satisfaction via postimplantation questionnaires; revision patients' satisfaction with the new large cylinder device as compared to their previous device; investigators' perception of what device they would have implanted if the large cylinders were not available.

## 2. Methods

### 2.1. Titan IPP

The Titan IPP is a hydraulic system designed to be surgically implanted into the penis for the management of ED. The implant provides the subject with voluntary control over the erect and flaccid states of the penis. The implant consists of two inflatable penile cylinders manufactured from Bioflex that are implanted in the corpora cavernosum of the penis. The cylinders are attached to the pump, which is placed in the subject's scrotum, and the pump is connected to a fluid reservoir that is implanted in the abdomen. The fluid reservoir is filled with a sterile saline solution. All components of the Titan are coated with a hydrophilic coating, a slick lubricious coating that rapidly absorbs aqueous solutions. The hydrophilic coating allows antibiotic solutions to be absorbed onto the IPP prior to implantation. 

### 2.2. EQM

The Fastsize EQM is a device that measures and monitors the strength of erections through a noninvasive pressure measurement. Erection strength is a measurement of penile axial rigidity. Measurements of 800 grams or more indicate a rigid penis. Measurements less than 500 grams indicate erections not strong enough for sexual intercourse involving penetration [7]. All subjects implanted with a Titan IPP with a cylinder size ≥24 cm were considered for enrollment. 

### 2.3. Subject Participation

To be included in the investigation, the subject must have met the following selection criteria: the subjects were males at least 18 years of age who received an implant or revised implant with a Titan IPP cylinder size ≥24 cm within the last year. In addition, the subject had to be informed of the nature of the study and agree to its provisions and provide written informed consent as approved by the Institutional Review Board. 

 Subjects were excluded based on the following criteria. The subject who was unable or unwilling to sign the Informed Consent Form was implanted with a cylinder size ≤22 cm or was unable to comfortably cycle the device at the time of enrollment. Written informed consent was obtained from all subjects. Enrollment and data collection were conducted at a follow-up visit at least 90 days and up to 1 year after implantation. 

### 2.4. Data Collected

At the follow-up visit (90 to 365 days post-implantation), medical history and demographic data were collected, along with operative data, the subject questionnaire, and rigidity measurements. Rigidity measurements were performed by subjects and study physicians with the FastSize EQM using the following procedure.

1. After ensuring the IPP was completely deflated, the subject was asked to inflate his device to a point where he thought it would be sufficient for sexual intercourse.
2. The number of pumps used to inflate the device was recorded.
3. The EQM was turned on by pressing the on/off switch.
4. The subject held the EQM unit in their primary hand and pushed the EQM pressure pad on the head of the penis.
5. The pressure pad was held for at least 5 seconds.
6. The output from the Fastsize EQM was recorded. If penis buckled, the output from the Fastsize EQM as the buckling force was recorded.
7. After the measurement was taken, the device was completely deflated.
8. The investigator repeated these steps for the investigator measurement of rigidity.

Standard summary statistics were calculated for all study variables. For continuous variables, statistics included means, standard deviations, and 95% confidence intervals for the means when normal distribution assumptions are not violated. Statistical analyses were conducted in SAS version 9.2 or above (SAS Institute, Cary, NC, USA). 

## 3. Results

A total of 17 patients were enrolled in the long cylinders study. Implantation dates ranged from 2/11/2009 to 10/16/2009 with baseline assessment dates from 9/21/2009 to 6/10/2010. All assessments were within the study-specified time range of 90 to 365 days after implantation (minimum 99 days, maximum 309 days, median 208 days). Of the 17 subjects enrolled, one did not meet inclusion criterion #3 (the subject received an implant or revised implant with a Titan IPP cylinder size of greater than or equal to 24 cm within the last year). No implant characteristics, rigidity measurements, or physician feedback data were available for this subject. Only the medical history and subject questionnaire data are available. These results are excluded from the summaries below. The demographic data (age, height, weight, and body mass index) are given in Table 1.

The etiology of ED among the study subjects, as well as presence of and information regarding Peyronie's disease, was noted (Table 2). Within this sample of patients, the most common etiology of ED was post-cancer treatment or vascular disease. Only 19% had peyronie's disease. 

Characteristics of the implants placed are described in Table 3. All implants were placed through a scrotal approach. Distal and proximal measurements were not provided in 88% of cases. Cylinder sizes used were primarily 24 and 26 cm (7 patients each size, 44%), with only 2 (12%) receiving 28 cm implants. Rear tip extension was not required for about half of the patients (9/16, 56%).

The physician feedback results are found in Table 4. Most physicians rated the final result as excellent (14/16, 88%), and the remaining two ratings were very good (2/16, 12%). With prior cylinders used, the mean rear tip extension utilized was 3.7 ± 1.4 cm. Only 3 of 16 subjects in this study were revisions patients (all previous implants were Titan). For the two revision subjects with data available on the size of the previous implant: one had a previous implant of 22 cm with 3 cm RTE and a new implant of 28 cm (no RTE); the other had a previous implant of 18 cm with 2 cm RTE and a new implant of 24 cm (no RTE).

Rigidity measurements are shown in Table 5. In general, the physicians performed a greater number of pumps to achieve perceived full inflation (*P* = 0.0003) than the patients and as a result achieved a higher rigidometer reading (*P* < 0.0001) and demonstrated less buckling than achieved when patients inflated the implants themselves.

Results for the subject satisfaction questionnaires are shown in Table 6 (initial implantation) and Table 7 (revisions). Although the number of subjects is small, patients with their first implant reported greater satisfaction than revision patients. However, among the nonrevision patients, a greater percentage did not find the implant soft enough to conceal when deflated. Nonrevision patients reported greater ease of inflation than deflation, as compared with revision patients. All patients (revision and nonrevision) responded with strong agreement or agreement with the statement that they were pleased with the hardness of their erections with inflation; yet, satisfaction, specifically with girth and length when inflated, was more varied among both groups of patients, with some (3/13, 23%) reporting disagreement (dissatisfaction) that the length was adequate, among nonrevision patients. 

## 4. Discussion

Results have varied among studies evaluating postoperative penile length, both flaccid [8] and erect, [9] and the relation to subjective treatment satisfaction among patients with IPP. Yet, the most common etiology of erectile dysfunction for a patient receiving an IPP is prostate cancer, and both radiation and radical prostatectomy have to be shown to shorten the penis [10, 11].

Various methods that affect patients' satisfaction with penile length following IPP surgery have been studied. These include penile lengthening by circumferential graft at the time of IPP placement [12]; external traction therapy [13]; PDE5 inhibitors and glans injection [14]; girth- and length-expanding cylinders [15]. In addition, surgical techniques, such as release of the suspensory ligament [16] and release of the penoscrotal web [17] have been shown to produce greater satisfaction with penile length following IPP placement. Since satisfaction is a very important aspect of any method of correcting sexual function, ways to improve patient satisfaction paramount in the treatment of erectile dysfunction. 

Traditionally, prosthetic urologists were taught to downsize or use a nonaggressive approach to measuring corpora length for choosing implant sizing, opting to choose smaller lengths when there was any question. This was done to prevent distal erosion with the commonly used semirigid rod implants. However, this is not a common occurrence with IPPs. Thus recently, Henry et al., reported a new length measurement technique that resulted in more patients being implanted with larger cylinders, with satisfaction rates comparable to standard measurement techniques [6]. Longer cylinders may give the patient a more natural appearing penis in erection and in flaccidity, because the RTE length is replaced by more cylinder length. Surgeons are finding that revision subjects are implanted with a larger size than their previous device. This can account for their original device expanding the corpora and creating a dynamic stretch over time, which results in increasing penile length [18]. 

With the new Titan XL cylinders, the prosthetic surgeon now has the ability to use more cylinder length and less RTE's for patients with the longer penis. The data show that in this prospective multicenter study, this new product can be successfully implanted as there were no infections and all IPPs cycled well at followup. In addition, the surgeons report no problems in implanting the new XL cylinders. 

The primary objective of this study was to assess the rigidity of the Titan IPP cylinders ≥24 cm using the Fastsize EQM postimplant. Secondary objectives included assessment of subjects' and investigators' perception of sufficient rigidity for sexual function; subject satisfaction via postimplantation questionnaires; revision patients' satisfaction with the new large cylinder device as compared to their previous device; investigators' perception of what device they would have implanted if the large cylinders were not available. The results show that the XL cylinders had great rigidity both objectively using the Fastsize EQM and subjectively by implanting surgeon questionnaire. These two results will hopefully quell the concern that longer cylinders may have too weak axial rigidity for sexual intercourse. Moreover, patient satisfaction rates with the XL cylinders were excellent too.

 Limitations to the study were that high-volume surgeons were used in this study and the average urologist may not have the same results. Another is that the patients were not randomized to receive the older version of what would have been implanted versus the new XL cylinders. A third limitation is that there were only 17 patients in the study, but these patients are only occasional, even at high-volume centers. Another limitation is that the study stopped enrolling after 17 patients because the Fastsize EQM was seized by the FDA in 2010 and states “The FastSize EQM Erectile Quality Monitor are misbranded and adulterated because they, among other things, are unapproved and were manufactured under conditions that did not meet current Good Manufacturing Practices (cGMP) requirements.”, and this calls into question the accuracy of this device.

## 5. Conclusion

 The new XL cylinders appear to give the prosthetic urologist the ability to use longer cylinders with apparently good patient satisfaction and great axial rigidity. Prosthetic urologists should feel more comfortable with placing longer cylinders (greater than 22 cm) in terms of minimal buckling when using the new XL cylinders and carefully showing the patient to apply more pumps during inflation. 

## Figures and Tables

**Table 1:** Demographic data of study subjects.

Characteristic	*n*	Mean ± SD	Median (Min, Max)	95% CI
Age at implant	16	66.8 ± 7.0	67.0 (53.6, 79.4)	63.0, 70.5
Height (cm)	16	180.9 ± 11.9	184.0 (142, 191)	174.5, 187.2
Weight (kg)	16	105.6 ± 14.0	106.5 (83, 126)	98.2, 113.1
Body mass index (kg/m^2^)	16	32.5 ± 5.0	32.5 (24.8, 41.7)	29.8, 35.2

Note: SD: standard deviation; CI: confidence interval.

**Table 2:** General ED etiology and presence of peyronie's disease.

Medical history condition	*n*/*N* (%)
Diabetes mellitus	1/16 (6%)
Vascular disease	6/16 (38%)
Pelvic surgery	0/16 (0%)
Pelvic trauma	0/16 (0%)
Post-cancer treatment	5/16 (31%)
Psychological causes	0/16 (0%)
Other	6/16 (38%)
Hypertension	2/16 (13%)
Malfunctioning penile prosthesis	3/16 (19%)
Severe impotence	1/16 (6%)
Peyronie's Disease	3/16 (19%)
Modeling conducted	2/3
Device maintained enough pressure to sufficiently model subject's anatomy	2/3

**Table 3:** Characteristics of implants assessed.

	*n*/*N* (%)
Implant approach/technique:	
Scrotal	16/16 (100%)
Infrapubic	0/16 (0%)
Right proximal measurement	
10 cm	2 (12%)
NA^1^	14 (88%)
Right distal measurement	
16 cm	2 (12%)
NA^1^	14 (88%)
Left proximal measurement	
10 cm	2 (12%)
NA^1^	14 (88%)
Left distal measurement	
15 cm	1 (6%)
16 cm	1 (6%)
NA^1^	14 (88%)
Cylinder size	
24 cm	7/16 (44%)
26 cm	7/16 (44%)
28 cm	2/16 (12%)
Right RTE	
None	9 (56%)
1 cm	5 (31%)
2 cm	2 (12%)
3 cm	0 (0%)
Left RTE	
None	9 (56%)
1 cm	6 (38%)
2 cm	1 (6%)
3 cm	0 (0%)
Reservoir size	
100 cc	15/16 (94%)
130 cc	1/16 (6%)

Note: ^1^NA: not available; no result given.

**Table 4:** Physician feedback.

	*n*/*N* (%)
Rate final result (5 = excellent, 1 = poor):	
5	14/16 (88%)
4	2/16 (12%)
3	0/16 (0%)
2	0/16 (0%)
1	0/16 (0%)
What device would you have previously used?	
Titan	15/16 (94%)
AMS CX	1/16 (6%)
AMS Ultrex/LGX	0/16 (0%)
AMS Ambicor	0/16 (0%)
What cylinder size?	
21	1/16 (6%)
22	14/16 (88%)
24	1/16 (6%)
What RTE size?	
*N *	16
Mean ± SD	3.7 ± 1.4
Median (Min, Max)	3.8 (2, 7)
95% CI	2.9, 4.4
Revision subject (yes)	3/16 (19%)
Previous device	
Titan	3
AMS CX	0
AMS Ultrex/LGX	0
AMS Ambicor	0
Cylinder size	
18 cm	1
22 cm	1
RTE size	
2 cm	1
3 cm	1

**Table 5:** Rigidity measurements.

	Patient	Physician
Number of pumps until perceiving full inflation		
*N *	16	16
Mean ± SD	30.4 ± 14.6	44.4 ± 8.3
Median (Min, Max)	28.5 (9, 58)	44.5 (33, 62)
95% CI	22.6, 38.2	40.0, 48.9
Rigidometer reading (g)		
*N *	16	16
Mean ± SD	1500.0 ± 342.5	1787.5 ± 212.5
Median (Min, Max)	1600 (800, 2000)	1800 (1400, 2000)
95% CI	1317.5, 1682.5	1674.3, 1900.7
Did the penis buckle?		
No (*n*/*N* (%))	5/16 (31%)	16/16 (100%)
Yes (*n*/*N* (%))	11/16 (69%)	0/16 (0%)

**Table 6:** Satisfaction of initial implantation patients.

How satisfied are you with the following aspects of your implant?	*N* = 13				
	Strongly agree	Agree	Neutral	Disagree	Strongly disagree
	*N* (%)	*N* (%)	*N* (%)	*N* (%)	*N* (%)
Overall function	8 (62%)	3 (23%)	2 (15%)	0 (0%)	0 (0%)
Soft enough to conceal when deflated	4 (31%)	4 (31%)	1 (8%)	4 (31%)	0 (0%)
Ease of deflation	4 (31%)	5 (38%)	3 (23%)	1 (8%)	0 (0%)
Ease of inflation	7 (54%)	6 (46%)	0 (0%)	0 (0%)	0 (0%)
Hardness of erection when inflated	11 (85%)	2 (15%)	0 (0%)	0 (0%)	0 (0%)
Girth when inflated	6 (46%)	3 (23%)	4 (31%)	0 (0%)	0 (0%)
Length when inflated	6 (46%)	2 (15%)	2 (15%)	3 (23%)	0 (0%)

**Table 7:** Satisfaction of revision patients.

Compared to your previous implant, how satisfied are you with the following aspects of your new large cylinder implant?	*N* = 3				
	Strongly agree	Agree	Neutral	Disagree	Strongly disagree
	*N* (%)	*N* (%)	*N* (%)	*N* (%)	*N* (%)
Overall function	1 (33%)	2 (67%)	0 (0%)	0 (0%)	0 (0%)
Soft enough to conceal when deflated	2 (67%)	1 (33%)	0 (0%)	0 (0%)	0 (0%)
Ease of deflation	1 (33%)	2 (67%)	0 (0%)	0 (0%)	0 (0%)
Ease of inflation	1 (33%)	1 (33%)	0 (0%)	1 (33%)	0 (0%)
Hardness of erection when inflated	1 (33%)	2 (67%)	0 (0%)	0 (0%)	0 (0%)
Girth when inflated	1 (33%)	1 (33%)	1 (33%)	0 (0%)	0 (0%)
Length when inflated	1 (33%)	1 (33%)	1 (33%)	0 (0%)	0 (0%)
